# A Psychoacoustic Feature Extraction and Spatio-Temporal Analysis Framework for Continuous Aircraft Noise Monitoring

**DOI:** 10.3390/s26061842

**Published:** 2026-03-14

**Authors:** Tianlun He, Jiayu Hou, Da Chen

**Affiliations:** 1 School of Precision Instrument and Opto-Electronics Engineering, Tianjin University, Tianjin 300072, China; lunqiqi@yeah.net; 2 School of Safety Science and Engineering, Civil Aviation University of China, Tianjin 300300, China; 2023095024@cauc.edu.cn; 3 Tianjin Engineering Research Center of Civil Aviation Energy Environment and Green Development, Civil Aviation University of China, Tianjin 300300, China

**Keywords:** aircraft noise monitoring, sensor-based signal processing, psychoacoustic features, spatiotemporal fusion, ADS-B data

## Abstract

Aircraft noise monitoring systems deployed at major airports typically rely on scalar energy-based indicators, which primarily describe integrated sound energy but provide limited representation of the spectral–temporal structure and perceptual attributes of aircraft noise. To address this limitation, this study proposes a sensor-based psychoacoustic feature extraction and spatiotemporal analysis framework for continuous aircraft noise monitoring under high-density operational conditions. An automatic noise monitoring system compliant with ISO 20906 was deployed to synchronously acquire acoustic waveforms and ADS-B trajectory data. A cascaded spatiotemporal fusion algorithm was developed to associate noise events with aircraft flight paths, followed by a model-stratified multidimensional IQR-based data cleaning strategy to suppress environmental interference and non-stationary outliers. Based on the cleaned dataset, a suite of psychoacoustic features—including loudness, sharpness, roughness, fluctuation strength, and tonality—was extracted to characterize the perceptual structure of aircraft noise beyond conventional energy metrics. Experimental results demonstrate that, under equivalent sound exposure levels, psychoacoustic features retain substantial discriminative information that is lost in scalar energy indicators. The coefficients of variation for fluctuation strength and tonality reach 43.2% and 22.1%, respectively, corresponding to 15–69 times higher sensitivity compared to traditional energy-based metrics. Furthermore, nonlinear manifold mapping using UMAP reveals clear topological separation between new-generation and legacy aircraft models in the psychoacoustic feature space, whereas severe overlap persists in energy-based representations. Correlation analysis further indicates decoupling between macro-level physical design parameters (e.g., bypass ratio, thrust) and perceptual feature dimensions, highlighting the limitations of energy-centric monitoring schemes. The proposed framework demonstrates the feasibility of integrating psychoacoustic feature extraction into continuous sensor-based aircraft noise monitoring systems. It provides a scalable signal processing pipeline for enhancing the resolution and interpretability of aircraft noise measurements in complex operational environments.

## 1. Introduction

Noise pollution, identified by the World Health Organization (WHO) as a significant environmental hazard, has emerged as a critical factor in environmental monitoring and public health evaluation [1,2]. Research has linked extended exposure to high noise levels to cardiovascular illness, cognitive decline, and persistent discomfort. Aircraft noise is recognized as a notably substantial factor in neighborhoods adjacent to major airports [3,4,5]. Among transportation noise sources, aircraft noise stands out due to its elevated sound pressure levels, a wide frequency spectrum, and significant non-stationary characteristics during flyover occurrences. The continually shifting spectral–temporal structure presents substantial barriers to precise characterization in unattended long-term airport noise monitoring systems. Recent studies have further shown that reliable aircraft-noise characterization in operational environments requires explicit linkage between measured acoustic data and flight-tracking information, particularly when multiple aircraft types and complex traffic conditions coexist [6,7,8].

Regulatory frameworks and policy evaluations increasingly highlight the significance of airport noise monitoring systems in evidence-based noise management and operational decision-making. EU Regulation 598/2014 stipulates noise-related operational restrictions at major airports and facilitates noise modeling via the Aircraft Noise and Performance (ANP) database, whereas the FAA’s 2021 Noise Policy Review underscores the growing dependence on real-time monitoring data in contemporary noise management practices [9,10,11,12]. Recent airport-focused studies also indicate that monitoring systems are increasingly being used not only for compliance reporting, but also for operational reconstruction, exposure mapping, and model validation under realistic traffic and meteorological variability [13,14].

In operational practice, airport noise monitoring systems primarily report regulatory energy-based indicators such as A-weighted sound level ($L_{A}$) and single-event exposure level ($L_{SE}$) [15,16]. While these metrics quantify overall noise intensity, they are limited in representing the spectral–temporal characteristics of broadband, non-stationary acoustic signals [17]. Traditional metrics prove inadequate for analyzing the refined spectral structure improvements delivered by modern high-bypass-ratio turbofan engines under real monitoring conditions. In practical monitoring scenarios, conventional energy-based metrics can provide a coarse separation between aircraft of different technological generations; however, their limited resolution results in overlapping energy distributions that obscure finer spectral and temporal differences in aircraft noise [18,19]. Merino- Martínez et al. [19] demonstrated through field measurements at Amsterdam Airport Schiphol that psychoacoustic metrics such as loudness, tonality, and psychoacoustic annoyance revealed significant differences between the Airbus A320 ceo and neo variants that were not captured by conventional energy indicators, underscoring the limitations of energy-centric evaluation for differentiating aircraft with similar certification noise levels. More recent perceptual studies have likewise emphasized that annoyance and quality judgments for single flyover events are better captured when psychoacoustic descriptors are considered alongside conventional level-based indicators [20,21].

Psychoacoustic models based on critical-band processing and auditory masking mechanisms provide a perceptually based alternative for defining sound quality beyond energy summaries. Metrics include loudness, sharpness, roughness, fluctuation strength, and tonality effectively encapsulate aspects of auditory experience that are relevant to noise annoyance but are not preserved in scalar energy indicators [22,23]. More recently, Lotinga et al. developed psychoacoustic prediction models for noise annoyance responses to unmanned aircraft systems (UAS), demonstrating that tonality and sharpness serve as significant second-order predictors beyond overall loudness [23]. In the domain of conventional aircraft, Soeta and Kagawa established a three-dimensional psychological evaluation structure relating aircraft noise perception to physical parameters [12]. Felix Greco et al. applied sound quality assessment to medium-range aircraft with enhanced fan-noise shielding, revealing perceptual improvements not fully reflected in certification-level metrics [18]. These studies collectively demonstrate the added value of psychoacoustic characterization; however, their application has been predominantly limited to laboratory simulations or short-duration field campaigns with controlled conditions. However, the stability and interpretability of these perceptual descriptors under continuous, unattended monitoring conditions—where multi-aircraft interference, trajectory ambiguity, and environmental contamination are prevalent—remain insufficiently examined [19,24,25,26].

Recent advances in sensor-based environmental noise monitoring—including IoT-enabled wireless acoustic sensor networks (WASNs), edge computing platforms, and multi-source data fusion architectures—have demonstrated the technical feasibility of continuous, large-scale noise data collection [27,28]. In the specific context of airport noise, Liu et al. proposed an IoT-based framework for airport noise perception monitoring combining multi-source data fusion with spatial distribution modeling [29]. Fu et al. developed a hybrid CNN–BiLSTM–Attention model integrating meteorological and trajectory data for aircraft noise level prediction [30]. Furthermore, psychoacoustic monitoring approaches using machine learning have been applied to building acoustic environments [31]. Ascari et al. examined the influence of traffic input data quality on noise estimation accuracy, illustrating the critical importance of data validation in sensor-based monitoring pipelines—a challenge directly relevant to the airport noise monitoring context [32]. Despite these advances in sensing infrastructure and predictive modeling, most systems remain focused on energy-level acquisition and do not extend the processing chain to perceptual-level feature extraction.

However, a systematic framework that integrates spatiotemporal data fusion, multidimensional data quality control, psychoacoustic feature extraction, and dimensionality reduction visualization within a continuous aircraft noise monitoring pipeline has not been established. Existing sensor-based monitoring studies primarily focus on energy-level acquisition and event detection, without extending the processing chain to include perceptual descriptors [33]. Conversely, psychoacoustic studies of aircraft noise typically operate on curated laboratory recordings or small-sample field measurements, without addressing the data reliability challenges inherent in continuous operational environments—such as multi-aircraft interference, ADS-B trajectory ambiguity, and environmental outlier contamination. Furthermore, existing research has not systematically examined the decoupling between aircraft physical design parameters (e.g., bypass ratio, maximum thrust, fan diameter) and perceptual feature dimensions under real monitoring conditions, leaving unexplained why aircraft noise events with similar energy levels can exhibit markedly different spectral and perceptual characteristics.

To address the limitations of conventional energy-based indicators in resolving the spectral–temporal structure of aircraft noise, this study proposes a continuous monitoring-oriented sound quality analysis pipeline built upon long-term, real-world airport data. Unlike previous psychoacoustic aircraft studies that primarily relied on laboratory recordings or short-term controlled field measurements, the proposed framework is specifically designed for unattended operational monitoring environments. To ensure methodological robustness under real-world conditions, a spatiotemporal association strategy integrating time-window alignment and spatial vector consistency verification is developed to link acoustic events with ADS-B flight trajectories. In contrast to conventional threshold-based event detection approaches, the proposed cascaded screening mechanism jointly evaluates acoustic features and trajectory plausibility, reducing multi-aircraft interference and environmental contamination prior to perceptual feature extraction.

Furthermore, rather than applying psychoacoustic metrics as post hoc descriptors of isolated events, the framework embeds perceptually grounded features within a data reliability-controlled monitoring pipeline, enabling aircraft-type-level comparative analysis under comparable exposure constraints. By explicitly examining the statistical dispersion and separability between traditional energy indicators and multidimensional psychoacoustic descriptors in continuous operational data, this study moves beyond laboratory-based sound quality evaluation and establishes a monitoring-compatible perceptual characterization approach for aircraft noise.

## 2. Materials and Methods

This chapter details the experimental methodology and data processing workflow adopted in this study for continuous aircraft noise monitoring. This includes the acquisition of raw data from continuous aircraft noise monitoring points, spatiotemporal coupling and data cleaning algorithms for heterogeneous datasets, and the computational principles of psychoacoustic feature metrics. By presenting the technical framework, from physical signal acquisition to acoustic feature extraction, this chapter establishes a reliable methodological basis for subsequent analysis of aircraft noise characteristics, feature distributions, and their relationships with physical design parameters.

### 2.1. Monitoring Instruments and Data Acquisition

#### 2.1.1. Monitoring Site Configuration

**Figure 1:**
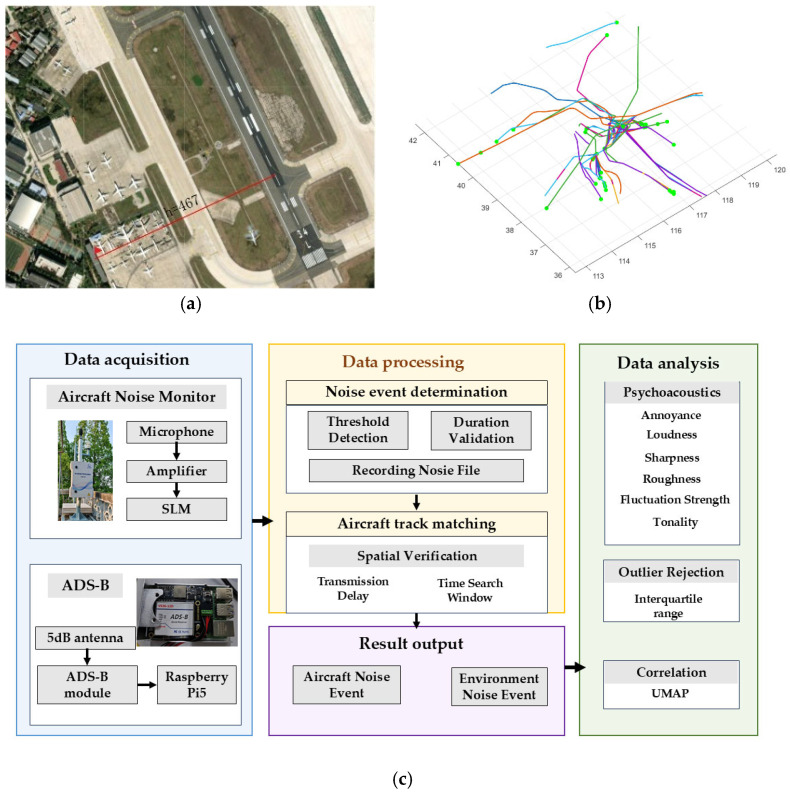
Integrated framework for continuous aircraft noise monitoring and flight trajectory association. (**a**) Geographic configuration of the monitoring site in relation to the runway environment. (**b**) Reconstructed three-dimensional flight tracks derived from ADS-B surveillance data. (**c**) System architecture. (Note: The Chinese characters on the equipment indicate it is an Automatic Aircraft Noise Monitoring Instrument).

This study was conducted at a major international airport equipped with dual parallel runways, representing a typical high-density flight operation environment. The acoustic measurement point was located atop a building approximately 450 m laterally from Runway 16R and about 6 m above ground level. This geometric configuration aligns, in horizontal projection, with the lateral noise certification reference point defined in ICAO Annex 16 Vol. I [34], as shown in Figure 1a.

The microphone position is vertically elevated over 1.2 m above the building roof, complying with ISO 20906 standards for automatic noise monitoring station deployment [35]. This rooftop placement strategy balances acoustic measurement accuracy with engineering feasibility. It effectively mitigates acoustic shadowing and multipath reflection effects caused by ground obstacles. Simultaneously, this location provides a suitable observation window for characterizing aircraft noise during the takeoff climb phase, when engines operate under near-maximum thrust conditions.

#### 2.1.2. Instrumentation and Signal Acquisition

This study employed the Feiyin 2400 aircraft noise monitoring system developed by Hangda Green Wing (Tianjin) Technology Co., Ltd. The system is equipped with a 1/2-inch prepolarized free-field microphone, providing a frequency response range of 20 Hz to 20 kHz and a dynamic range of 20–140 dB. Acoustic signals are digitized using 24-bit analog-to-digital conversion at a sampling rate of 48 kHz.

Noise event triggering follows the detection methodology specified in ISO 20906. Once predefined thresholds are exceeded, the system automatically records complete time-domain waveforms and derives key acoustic parameters, including peak A-weighted sound level, event duration, and SEL (Sound Exposure Level) within the $L_{Amax}-10\;\mathrm{dB}$ interval.

In parallel with acoustic acquisition, aircraft trajectory information is obtained through an integrated 1090 MHz ADS-B receiving chain. ADS-B broadcast messages are continuously decoded in real time, and essential flight parameters—such as ICAO address, flight identifier, latitude, longitude, altitude, and timestamp—are extracted and transmitted to the central processing server. This design enables precise spatiotemporal association between aircraft kinematics and measured acoustic events.

To enhance the controllability and reproducibility of the sensing pipeline, the ADS-B reception module is implemented as a Raspberry Pi-based edge node connected to the 1090 MHz receiver front-end. The modular edge architecture supports stable data streaming and flexible deployment in long-term monitoring scenarios.

### 2.2. Spatiotemporal Association and Data Validation

#### 2.2.1. Noise Event Matching Algorithm Based on Spatiotemporal Coupling Mechanism

Due to the close proximity of the monitoring site to the runway system, multiple aircraft are frequently present within overlapping acoustic influence regions. Under such traffic density, direct one-to-one timestamp alignment between acoustic detections and ADS-B trajectories may result in ambiguous or incorrect associations. To improve reliability, a cascaded spatiotemporal validation mechanism was introduced, consisting of a coarse temporal screening stage followed by a geometric consistency verification.

1. Time-Domain Coarse Screening Based on Transmission Delay Characteristics.

The acoustic monitoring terminal and the ADS-B receiver operate through independent data acquisition and communication paths, each introducing its own processing and transmission latencies. Specifically, once a noise event is triggered, the acoustic system must complete waveform recording over the full event duration, perform on-board parameter computation, and transmit the packaged record to the central server. Meanwhile, the ADS-B decoding chain involves message buffering at the edge node and periodic batch uploading. Although both subsystems are referenced to a unified time base through combined NTP synchronization and GPS timing, residual clock offsets and variable processing delays are unavoidable.

To accommodate these cumulative asynchronous effects, a symmetric temporal search window of ±60 s centered on the acoustic event trigger time $T_{event}$ was adopted for trajectory matching. The selection of this threshold is supported by two considerations. First, empirical synchronization statistics collected during the commissioning phase indicated that all valid acoustic–trajectory correspondences were consistently confined within this interval. Second, operational analysis of the local airport traffic flow shows that the temporal separation between two consecutive aircraft movements at the monitored runway is no less than 90 s under standard scheduling conditions. Therefore, a ±60 s window ensures sufficient tolerance for network and processing delays while maintaining unambiguous trajectory association, effectively avoiding cross-matching between adjacent flights.

Set the trigger time of any captured noise event as $T_{event}$. Based on this, establish a time tolerance threshold τ=60 s to generate a symmetric matching window $\Omega_{t}$. The system performs retrospective and prospective searches within the ADS-B track database:

(1) $$T_{track}\in \left[T_{event}-\tau ,\;T_{event}+\tau \right]$$

Among these, $T_{track}$ represents the nearest neighbor timestamp of the candidate aircraft track. Only when the track data falls within this confidence interval centered around the noise is the track marked as a “primary correlated track,” thereby completing the Temporal Correlation Locking from acoustic events to kinematic trajectories.

2. Spatial Geometric Verification Based on Flight Path Vector Features.

To eliminate interference from adjacent airspace (such as parallel runway approaches or overflights in non-target airspace), spatial geometric topology constraints are introduced at this stage. Considering that the monitoring site is located approximately 450 m laterally from the runway centerline and close to the runway threshold, aircraft generating detectable noise events at this location are typically in a relatively low-altitude phase of flight. Therefore, the algorithm focuses on trajectory segments with H<300 m, to ensure that only flight tracks physically relevant to the monitored overflight events are considered.

Within this low-altitude subset, the start and end coordinate vectors of the trajectory points are extracted, and refined discrimination is performed by calculating the vector deviation between the track heading angle and the physical orientation of the runway.

Trajectory Differential Increment Solution: Let the coordinates of the start and end points of the trajectory segment be $P_{start}\left(\phi_{1},\lambda_{1}\right)$ and $P_{end}\left(\phi_{2},\lambda_{2}\right)$, where ϕ denotes latitude and λ denotes longitude. Calculate the differential increment of the trajectory in spherical coordinates:

(2) $$\Delta \phi =\phi_{2}-\phi_{1}$$

(3) $$\Delta \lambda =\lambda_{2}-\lambda_{1}$$

Kinematics Azimuth Angle Mapping: Based on the Mercator projection principle, calculate the azimuth angle $\alpha_{track}$ of the aircraft’s instantaneous motion vector, with true north as the reference baseline:

(4) $$\alpha_{track}=arctan\left(\frac{\Delta \lambda \cdot \cos\phi_{mean}}{\Delta \phi }\right)$$

where $\cos\phi_{mean}$ represents the correction factor for latitude-related distortion, improving the accuracy of longitude scaling.

Vector consistency determination: Define the physical heading of the target runway as $\alpha_{runway}$, and construct a deviation function D(α):

(5) $$D\left(\alpha \right)=\left|\alpha_{track}-\alpha_{runway}\right|$$

Set the tolerance threshold ϵ=30°. If $D\left(\alpha \right)<\epsilon$ the track direction is considered consistent with the runway orientation, confirming a valid target event; otherwise, it is classified as a spatial outlier and discarded.

#### 2.2.2. Non-Parametric Outlier Rejection Based on Stratified

Because the subsequent analysis focuses on comparing statistical characteristics among different aircraft types, the outlier rejection procedure must avoid introducing bias across categories. Applying a single global threshold to the entire dataset may incorrectly classify naturally quieter or louder aircraft as anomalies, thereby distorting inter-type comparisons.

To ensure fairness and statistical stability, the IQR (interquartile range) method is applied separately within each aircraft type. This stratified strategy preserves intrinsic differences among models while removing abnormal measurements that are inconsistent with the typical distribution of the corresponding category. In addition, the non-parametric nature of the IQR approach makes it suitable for aircraft noise data, which often exhibit skewed or heavy-tailed characteristics under real operational conditions.

For an univariate variable X, the first quartile $Q_{1}=P_{25}(X)$, third quartile $Q_{3}=P_{75}(X)$, and interquartile range is defined as $IQR=Q_{3}-Q_{1}$. The outlier boundaries are then given by:

(6) $$L_{b}=Q_{1}-k\cdot IQR$$

(7) $$U_{b}=Q_{3}+k\cdot IQR$$

When an observation $x_{i}$ satisfies either $x_{i}<L_{b}$ or $x_{i}>U_{b}$, it is classified as an outlier. This study employs k=1.5 as the threshold for identifying “mild outliers,” striking a balance between removing obvious anomalies and preserving data representativeness. Because aircraft types exhibit different baseline noise distributions, applying a global threshold across all records may introduce bias in subsequent comparisons. Therefore, the IQR procedure is performed independently within each aircraft type.

The filtered dataset is divided into 15 subsets based on model type $g\in \{g_{1},g_{2},...,g_{10}\}$, with each subset containing all observations for the same model type. For each model type g and noise metric $m\in \{{Lepn}_{max}\}$, the quartile is calculated internally for that model type:

(8) $$Q_{1}^{\left(g,m\right)},Q_{3}^{\left(g,m\right)},IQR^{\left(g,m\right)}=Q_{3}^{\left(g,m\right)}-Q_{1}^{\left(g,m\right)}$$

Corresponding outlier boundaries:

(9) $$L_{b}^{\left(g,m\right)}=Q_{1}^{\left(g,m\right)}-1.5\cdot IQR^{\left(g,m\right)}$$

(10) $$U_{b}^{\left(g,m\right)}=Q_{3}^{\left(g,m\right)}+1.5\cdot IQR^{\left(g,m\right)}$$

For observation $x_{i}$ in model g, a multidimensional joint constraint criterion is applied: if any single metric exceeds the model’s threshold, the entire record is flagged as an outlier and discarded.

(11) $$Outlier(x_{i})=\underset{m}{⋃}\left\{x_{i}^{\left(m\right)}<L_{b}^{\left(g,m\right)}\}\cup \{x_{i}^{\left(m\right)}>U_{b}^{\left(g,m\right)}\right\}$$

### 2.3. Psychoacoustic Evaluation Model Based on Auditory Perception Mechanisms

Traditional acoustic metrics have limitations in describing the complex characteristics of broadband and non-stationary aircraft noise signals. This study introduces a psychoacoustic analysis framework derived from critical-band processing and masking mechanisms, enabling a quantitative transformation from physical acoustic measurements to auditory-related feature representations. The core parameters employed in this study are defined as follows:

#### 2.3.1. Loudness (N)

Loudness characterizes the nonlinear subjective perception of sound energy intensity by the human ear, measured in sones (sone). Unlike simple sound pressure levels, the loudness model incorporates corrections for the human ear’s equal loudness contours across different frequencies and the spectral masking mechanism between frequency bands. This study follows the ISO 532-1 standard [36] and employs the Zwicker fixed model. Total loudness is calculated by integrating characteristic loudness over the critical frequency band rate.

(12) $$N=\int_{0}^{24Bark}N^{\prime }(z)dz$$

Here, z denotes the critical frequency band rate, with the integration interval covering the 0–24 Bark frequency domain of human auditory perception.

#### 2.3.2. Sharpness (S)

Sharpness quantifies the “harsh” or “piercing” sensation produced by high-frequency components in the acoustic spectrum, measured in acum units. This metric essentially reflects the position of the center of mass in the loudness spectrum. The computational model incorporates a frequency weighting function g(z) to perform weighted moment integration on high-frequency loudness characteristics, normalized using total loudness:

(13) $$S=0.11\cdot \frac{\int_{0}^{24Bark}N^{\prime }(z)\cdot g(z)\cdot zdz}{\int_{0}^{24Bark}N^{\prime }(z)dz}$$

#### 2.3.3. Roughness (R)

Roughness describes the discontinuous, coarse texture induced by fast amplitude modulation $\left(15\;\mathrm{Hz}<f_{mod}<300\;\mathrm{Hz}\right)$ in sound, measured in asper (asper). This perception primarily arises because energy fluctuations within the critical frequency band cannot be smoothed by the auditory system’s temporal integration. Based on the Zwicker & Fastl model, roughness R exhibits a nonlinear relationship with modulation frequency $f_{mod}$ and modulation depth ∆L within a specific frequency band:

(14) $$R=c_{cal}\cdot \int_{0}^{24Bark}f_{mod}\left(z\right)\cdot ∆L(z)dz$$

In the equation, $c_{cal}$ represents the calibration constant. For turbofan engines, the blade passing frequency (BPF) and its harmonics constitute the primary acoustic source generating roughness.

#### 2.3.4. Fluctuation Strength (F)

Fluctuation characterizes the “undulating” or “oscillating” sensation produced by slow amplitude modulation in sound, measured in vacils (vacil). Unlike roughness, fluctuation corresponds to temporal envelope variations discernible to the human ear. Its calculation logic shares origins with roughness but emphasizes low-frequency modulation components:

(15) $$F=\frac{0.008\cdot \int_{0}^{24Bark}∆L(z)\cdot dz}{\left(\frac{f_{mod}}{4Hz}\right)+(\frac{4Hz}{f_{mod}})}$$

The Doppler effect in aviation noise and fluctuations in sound propagation caused by atmospheric turbulence are the primary contributors to waviness.

#### 2.3.5. Tonality (T)

Tonal quality aims to quantify the prominence of discrete spectral components in mixed noise, serving as a key factor in eliciting subjective annoyance levels. This study employs the Aures frequency-domain model, which calculates the degree to which pure-tone components exceed the masking threshold relative to background noise:

(16) $$T=C\cdot \sum_{i}{\left[\omega_{i}\cdot ∆L_{i}\right]}^{p}$$

In the equation, $∆L_{i}$ denotes the sound level difference by which the i-th pure-tone component exceeds the critical band masking threshold, while $\omega_{i}$ represents the frequency-dependent perceptual weighting coefficient. This metric accurately captures the monophonic characteristics of turbofan engines under high-speed operating conditions.

## 3. Results

This section presents the analysis results derived from the validated aircraft noise event dataset obtained through the continuous monitoring system. The objective is to examine how different feature representations describe aircraft noise characteristics under operational conditions.

The results are organized into several aspects, including dataset consistency verification, comparative behavior between conventional energy-based metrics and psychoacoustic features, variability under similar energy levels, and the distribution patterns of aircraft-related acoustic attributes in the multidimensional feature space. These analyses aim to evaluate the descriptive capability of the adopted feature framework and its effectiveness in differentiating aircraft models.

### 3.1. Noise Event Matching and Dataset Construction

#### 3.1.1. Spatiotemporal Coupling Matching for Heterogeneous Data

In continuous aircraft noise monitoring, reliable association between acoustic events and flight trajectories is essential for constructing a valid analysis dataset. Following the spatiotemporal matching strategy described in Section 2.2, the algorithm was applied to data collected in a high-density operational environment.

During the monitoring period, a total of 1465 acoustic noise event files were recorded by the terminal. A unified time reference was established through the combined use of NTP synchronization and GPS timing, minimizing clock drift among sensing components. Because the acoustic terminal and ADS-B receiver transmit data through independent communication paths, timestamp offsets remain unavoidable. Therefore, each detected event with trigger time $T_{\mathrm{event}}$ defined a symmetric search window of ±60 s, within which candidate trajectories were retrieved from the ADS-B database.

Using this temporal criterion, 1271 noise events were successfully associated with at least one potential aircraft trajectory, forming the initial temporally matched dataset. The remaining 194 events had no corresponding ADS-B trajectory within the defined time window and the predefined geographic query range centered on the monitoring site. In other words, no flight track data were available within the relevant spatiotemporal domain for these events, indicating that no aircraft trajectory was present within the effective matching range during the corresponding acoustic trigger interval.

However, temporal agreement alone cannot fully remove interference from aircraft operating in adjacent airspace, such as parallel runway approaches or non-target overflights. Therefore, spatial verification based on low-altitude flight segments (H < 300 m) was further performed. The motion direction of each candidate track was compared with the physical orientation of the runway. When the heading deviation satisfied D(α)<30°, the association was accepted; otherwise, the event was rejected.

After applying this geometric filtering, the number of valid samples was reduced from 1271 to 1078, indicating that the spatial constraint effectively removed false associations while preserving the majority of relevant events.

To assess the robustness of the spatiotemporal matching strategy, a sensitivity analysis was conducted by varying the temporal window (±30 s, ±45 s, ±60 s, ±90 s) and the heading deviation threshold (15°, 30°, 45°). Using the manually verified set of 1056 aircraft events as reference, each configuration was evaluated in terms of precision and recall (Table 1).

Restricting the temporal window to ±30 s resulted in reduced recall (79.55–90.91%), indicating under-coverage of valid overflight events despite high precision (≈95–96%). Expanding the window to ±45 s substantially improved recall (up to 97.54%) while maintaining precision above 94%. Under the ±60 s condition, the 30° angular threshold achieved full recall (100%) with high precision (97.96%), corresponding to only 22 false positives. Tightening the angular constraint reduced recall, whereas relaxing it increased false associations. When the window was extended to ±90 s, recall remained high but precision declined (down to 90.26% at 45°), reflecting increased incidental temporal overlap.

Overall, the ±60 s and 30° configuration provides the most balanced trade-off, preserving all 1056 validated events while limiting false matches. This setting was therefore adopted as the optimal spatiotemporal matching parameter combination in this study.

After manual verification to remove environmentally contaminated or trajectory-inconsistent cases, the final dataset consisted of 1056 validated aircraft overflight events, demonstrating strong consistency between the acoustic monitoring system and ADS-B trajectory data.

#### 3.1.2. Outlier Removal

Among the 1078 spatially validated events, several aircraft categories contained only a small number of observations, which limited the reliability of statistical comparisons. To ensure stability in subsequent analyses, a minimum sample size requirement of N>25 was applied at the aircraft-type level.

After this screening, 15 major aircraft categories were retained, resulting in a working dataset of 738 events. The retained set covers the primary fleet operating in the monitored airspace and provides sufficient representation for comparative evaluation.

**Figure 2:**
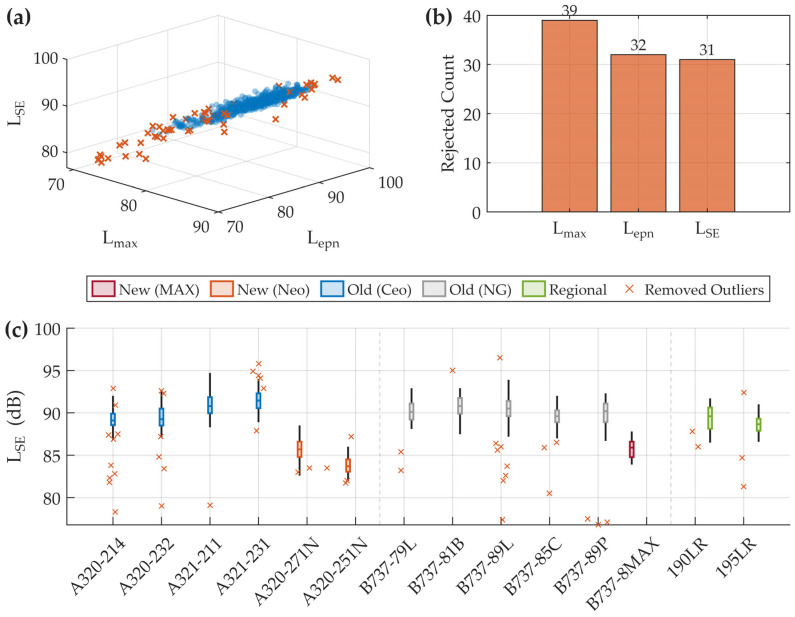
Validation of the dataset after multidimensional IQR filtering. (**a**) Distribution of samples in the Lmax-Lepn-LSE feature space, where blue markers denote retained events and red markers indicate removed outliers. (**b**) Contribution of each acoustic metric to the outlier identification process. (**c**) Distribution of cleaned data across aircraft categories, with excluded observations shown as red crosses. The dashed lines denote aircraft series of various airlines.

To further reduce the influence of abnormal measurements and environmental fluctuations, a model-specific multidimensional IQR filtering procedure was conducted. Within each aircraft category, statistical limits were computed for key metrics including $L_{max}$, $L_{epn}$ and $L_{SE}$ Figure 2a presents the distribution of samples in the feature space defined by these indicators. Observations exceeding the upper bound $Q_{3}+1.5\mathrm{IQR}$ were removed, as illustrated in Figure 2b. After applying these steps, the remaining dataset exhibited improved compactness and consistency, providing a reliable basis for the subsequent comparative and structural analyses.

As shown in Figure 2a, the rejected samples (red markers) are mainly distributed around the outer boundary of the main data cluster, indicating that these observations deviate from the dominant statistical structure of aircraft noise events. Figure 2b summarizes the contribution of each acoustic metric to the outlier identification process. Among them, $L_{max}$ shows the highest sensitivity, followed by $L_{epn}$ and $L_{SE}$. This suggests that instantaneous peak levels are more likely to be influenced by sporadic disturbances, supporting the use of multidimensional constraints in improving dataset stability.

The overall effectiveness of the filtering strategy is further illustrated in Figure 2c. By overlaying the excluded observations onto the model-wise distributions, it can be seen that abnormal points are removed while the internal statistical spread of each aircraft category remains largely preserved.

After this procedure, 53 records were excluded, leaving 685 valid samples for subsequent analysis. Detailed aircraft category information is summarized in Table 2.

### 3.2. Associations Between Aircraft Configuration Parameters and Acoustic Metrics

To examine how aircraft configuration variables relate to acoustic observations, an association analysis was conducted between physical parameters and both traditional energy metrics and psychoacoustic descriptors.

Spearman’s rank correlation coefficient was adopted to quantify monotonic relationships, and only statistically significant links (*p* < 0.05) were retained. The analyzed parameters include bypass ratio, thrust, fuselage length, wingspan, maximum takeoff weight, and aircraft age.

Rather than implying causality, this analysis provides a comparative view of how different acoustic metrics respond to variations in aircraft characteristics, thereby offering contextual support for the necessity of multidimensional feature representations.

**Figure 3:**
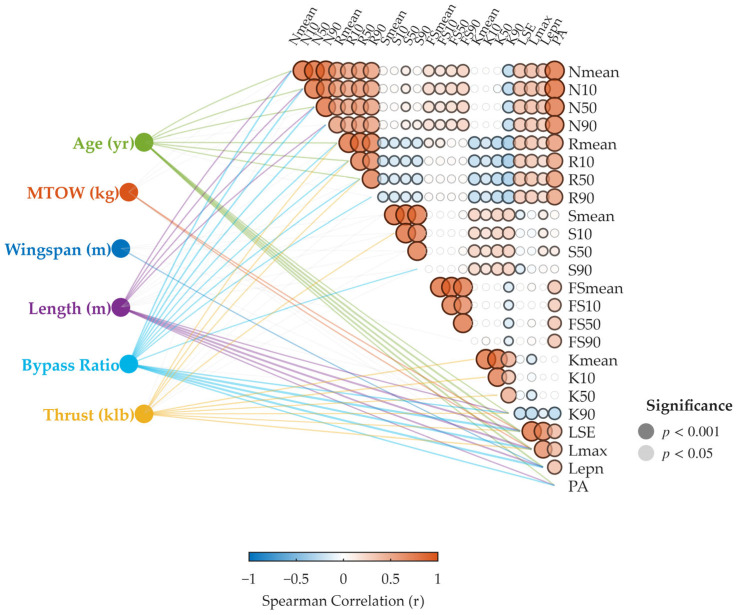
Correlation network between aircraft configuration parameters and acoustic metrics, together with the collinearity matrix of acoustic descriptors. Left nodes denote physical parameters, while right nodes represent acoustic indicators. Line color and thickness encode the magnitude and direction of Spearman’s correlation coefficient. Only statistically significant links (p<0.05) are displayed. The triangular matrix on the right illustrates internal cross-correlations among acoustic metrics.

As illustrated in Figure 3, the association results are presented as a correlation network. Nodes on the left correspond to aircraft configuration parameters, including thrust, bypass ratio, fuselage length, wingspan, maximum takeoff weight, and age. Nodes on the right represent acoustic descriptors, arranged from energy-based metrics toward psychoacoustic features.

The color and thickness of the connecting lines encode the sign and magnitude of Spearman’s correlation coefficient (ρ), where red denotes positive association and blue denotes negative association. Bubble size and shading reflect statistical significance, with darker tones indicating lower *p*-values. The color bar summarizes the correlation range from −1 to +1.

The map reveals heterogeneous response patterns: some metrics show relatively strong associations with certain aircraft parameters, whereas others remain weakly related. This diversity indicates that different descriptors capture complementary aspects of aircraft noise, motivating the inclusion of psychoacoustic dimensions in addition to energy measures.

As illustrated in Figure 3, aircraft configuration parameters exhibit clear associations with traditional energy-based metrics, while their relationships with psychoacoustic descriptors appear substantially weaker.

The bypass ratio shows consistent negative correlations with energy quantities such as $L_{max}$, $L_{epn}$, and $L_{SE}$. These links are among the strongest observed in the network, suggesting that propulsion architecture remains a primary factor influencing the overall magnitude of radiated acoustic energy. In contrast, connections between bypass ratio and perceptual metrics (e.g., roughness, sharpness, tonality) are sparse and generally weak, implying that reductions in total sound energy do not necessarily translate into proportional changes in perceived sound character.

A different pattern is observed for thrust, maximum takeoff weight, and fuselage length. These parameters tend to show positive associations with energy indicators, reflecting the intuitive tendency for larger or more powerful aircraft to generate higher absolute sound levels. However, similar to the bypass ratio, their relationships with psychoacoustic quantities are limited in both density and magnitude. Only occasional weak correlations are observed for certain descriptors, and no uniform trend emerges across perceptual dimensions.

Wingspan and aircraft age demonstrate comparatively modest relationships with both metric families. Although some links with energy indicators are present, their explanatory contribution is secondary relative to propulsion-related parameters. In the perceptual domain, most correlations remain weak or statistically marginal.

Overall, a notable structural contrast emerges: physical parameters align more strongly with sound energy than with sound quality.

This mismatch indicates that while macroscopic design variables are effective predictors of “how loud” an aircraft is, they provide incomplete information regarding how the sound is perceived. Psychoacoustic metrics, which depend on spectral distribution, temporal modulation, and auditory filtering mechanisms, appear to capture dimensions that are not linearly governed by global geometric or performance parameters.

The collinearity matrix on the right further supports this interpretation. Strong dependencies are observed within different statistical representations of the same attribute, whereas cross-category coupling between energy and perceptual descriptors is comparatively limited. Such a structure suggests that energy and perception occupy related but non-identical subspaces of the acoustic feature domain.

These observations provide quantitative motivation for extending aircraft noise evaluation beyond single-number energy metrics toward multidimensional perceptual representations.

### 3.3. Comparative Analysis of Traditional Energy Metrics and Acoustic Quality Metrics

Following the construction of a validated dataset and the extraction of multidimensional acoustic descriptors, this section examines how different metric systems organize aircraft noise information in feature space.

Conventional assessment frameworks predominantly rely on energy-related quantities such as maximum sound level, exposure level, and integrated sound pressure measures. These indicators provide compact summaries of acoustic magnitude and are widely used in regulatory and operational contexts. However, the extent to which such scalar descriptors preserve the structural variability embedded in complex, non-stationary aircraft noise remains an open question.

To explore this issue, a manifold-learning approach based on Uniform Manifold Approximation and Projection (UMAP) is employed. The objective is not dimensionality reduction per se, but the visualization of intrinsic data geometry under different metric representations.

Two feature domains are constructed:

- An energy-metric space, composed of $L_{max}$, $L_{epn}$, and $L_{SE}$;
- A psychoacoustic feature space, containing perceptually motivated descriptors derived from auditory modeling.

By projecting each space onto a two-dimensional manifold, the method enables direct comparison of clustering compactness, overlap patterns, and relative separability among aircraft samples.

If a metric system captures meaningful structural differences, clearer aggregation patterns and more stable neighborhood relationships are expected to emerge in the embedded space. Conversely, strong overlap would suggest limited discriminative capacity.

**Figure 4:**
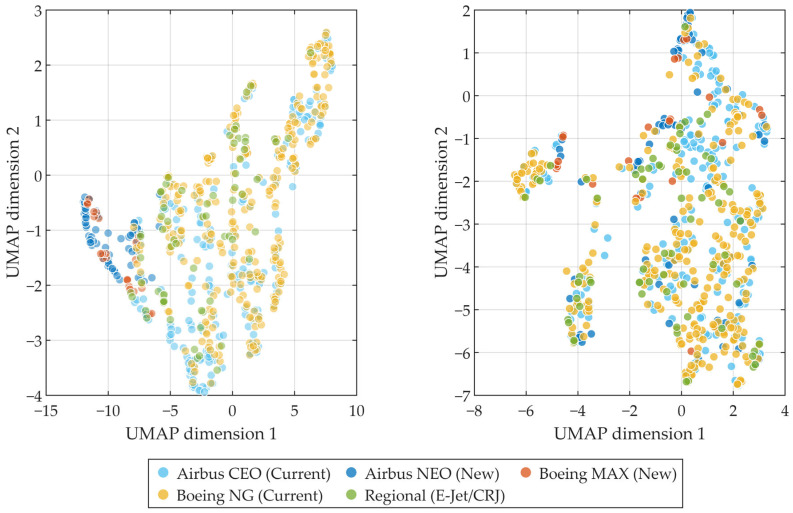
Comparative manifold representations of aircraft noise features obtained using UMAP. Left: Two-dimensional embedding constructed from traditional energy-based metrics ($L_{max}$, $L_{epn}$, $L_{SE}$). Right: Two-dimensional embedding constructed from multidimensional psychoacoustic sound quality metrics.

The comparative visualization results are presented in Figure 4.

As shown in Figure 4, the manifold derived from traditional energy metrics was obtained using UMAP (n_neighbors = 15, min_dist = 0.1, Euclidean metric, fixed random seed). The embedding exhibits a dominant global gradient associated with overall sound level, while aircraft from different technical families remain largely intermixed.

Quantitative analysis confirms this structural concentration. As summarized in Table 3, the first two principal components of the energy descriptors explain 98.38% of the total variance, and the participation ratio dimension is only 1.20. This indicates that the energy feature space is effectively constrained to a near one-dimensional manifold dominated by amplitude scaling, with limited preservation of higher-order spectral or temporal variability.

In contrast, the psychoacoustic manifold in Figure 4 displays a more diversified spatial organization. Although local coherence is preserved, the data unfold along multiple independent directions. Table 3 shows that the first two principal components account for only 54.98% of the variance, and the participation ratio dimension increases to 4.62, indicating substantially higher intrinsic dimensionality. These results suggest that psychoacoustic descriptors retain additional perceptual degrees of freedom related to spectral balance, modulation, and masking behavior.

To verify robustness against hyperparameter choices, n_neighbors (10–30) and min_dist (0.05–0.2) were systematically varied. Neighborhood stability was evaluated using mean Jaccard similarity of k-nearest neighbor sets. As reported in Table 4, the energy embedding achieves 0.766 ± 0.027, while the psychoacoustic embedding yields 0.706 ± 0.033. Both values indicate stable topology across parameter configurations. The slightly lower stability for psychoacoustic features is consistent with their higher intrinsic dimensionality, reflecting increased structural flexibility rather than instability. Full sweep results are provided in Supplementary Table S1.

Overall, the combined intrinsic dimensionality and robustness analyses demonstrate that psychoacoustic descriptors reorganize the dataset along multiple effective axes while maintaining stable manifold structure, providing a structurally richer representation than scalar energy metrics.

### 3.4. Experimental Comparison of Perceptual Feature Divergence Characteristics Under Equal-Energy Conditions

Following the manifold observations, this section formally examines whether psychoacoustic descriptors retain variability under equal-energy constraints. Conventional assessment frameworks frequently rely on integrated quantities such as the sound exposure level $L_{SE}$, implicitly assuming that events with comparable energy produce comparable auditory impressions. To evaluate this assumption, an equal-energy constrained analysis was performed.

**Figure 5:**
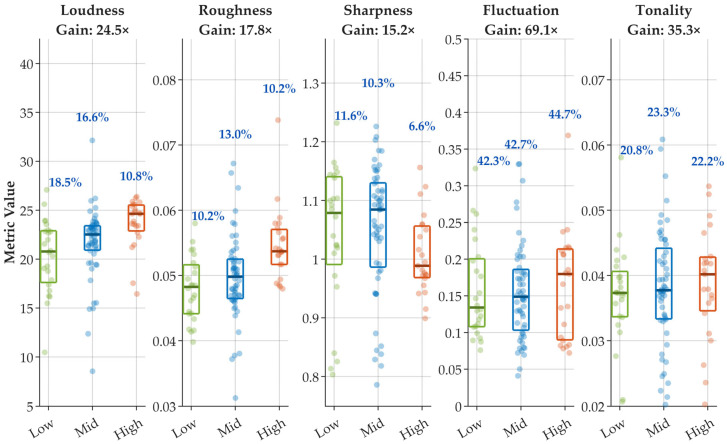
Distribution of psychoacoustic descriptors for the B737-89L under three narrow sound exposure level ($L_{SE}$) intervals (Low, Mid, High). Data points and boxplots are color-coded based on the $L_{SE}$: green for low, blue for medium, and orange-red for high levels.

The dataset was partitioned into three narrow $L_{SE}$ intervals, thereby limiting energy variability to a minimal range. For the representative B737-89L model (Figure 5), each bin contains 23–56 events. As summarized in Table 5, the residual dispersion of the controlling energy metric remains low, with coefficients of variation between 0.60% and 0.66%, confirming effective energy control within each interval.

Despite this stringent constraint, several psychoacoustic descriptors exhibit substantially larger variability. Fluctuation strength shows coefficients of variation between 42.3% and 44.7% (95% bootstrap CI approximately 31–56%), while tonality ranges from 20.8% to 23.3% (95% CI approximately 11–28%). Loudness and roughness remain between 10% and 18%, consistently exceeding the residual variation of $L_{SE}$. The detailed dispersion values, confidence intervals, and sample sizes for each bin are reported in Table 5.

To quantify relative sensitivity, we define Gain = CV_metric/CV_LSE. Gain ranges from approximately 11× (sharpness) to over 74× (fluctuation strength). Bootstrap ratio testing confirms that Gain is significantly greater than unity in all cases (*p* < 0.001), indicating that perceptual dispersion systematically exceeds residual energy dispersion under equal-energy control.

These results demonstrate that aircraft noise events with nearly identical integrated energy may still differ considerably in their fine temporal and spectral characteristics. Rather than contradicting energy-based evaluation, this finding highlights that psychoacoustic descriptors preserve complementary variability that is compressed when signals are reduced to a single scalar energy level.

**Figure 6:**
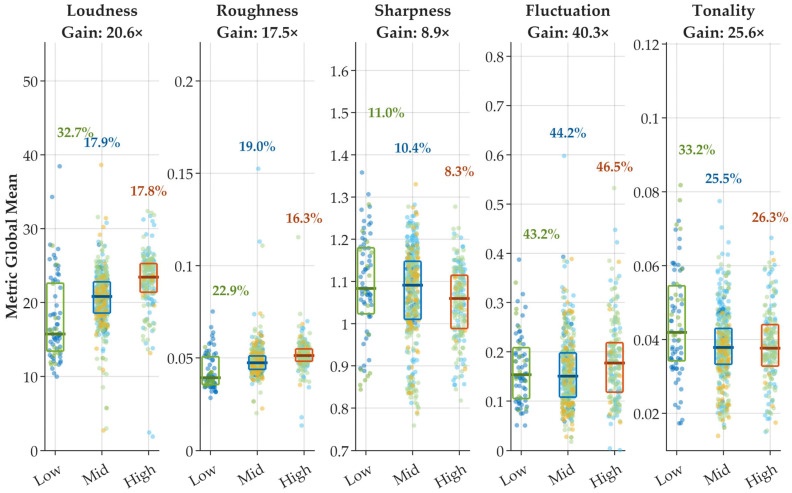
Distribution of psychoacoustic descriptors for the full dataset under three narrow sound exposure level ($L_{SE}$) intervals (Low, Mid, High). The multi-colored data points represent different aircraft categories (e.g., MAX, Neo, CEO, etc.).

To examine whether the divergence observed in the single-model analysis persists at a broader scale, the study further extends the equal-energy constrained evaluation to the aggregated dataset comprising multiple aircraft categories, as presented in Figure 6.

As the sample size increases to the full dataset, the empirical distributions of psychoacoustic metrics become statistically smoother, and extreme values appear less pronounced. Nevertheless, substantial dispersion remains evident across all perceptual descriptors.

Table 6 summarizes the coefficient of variation (CV), bootstrap-based 95% confidence intervals, and dispersion gain (CV_metric/CV_LSE) under equal-energy constraints. Across the three energy bins (Low: *n* = 73; Mid: *n* = 407; High: *n* = 195), the residual variability of the controlling energy metric L_SE remains low (CV_LSE ≈ 0.87–1.24%).

In contrast, psychoacoustic descriptors exhibit markedly higher dispersion. For example:

- Fluctuation Strength shows CV values of 43.2% (Low), 44.2% (Mid), and 46.5% (High), corresponding to gain factors of 34.8×, 36.4×, and 53.8×, respectively.
- Loudness maintains CV values between 17.8% and 32.7%, with gain ranging from 14.8× to 26.3×.
- Tonality presents gains between 21.0× and 30.4×.
- Even the most stable descriptor, Sharpness, yields gains between 8.6× and 9.5×.

Importantly, the 95% bootstrap confidence intervals of Gain do not overlap unity in any case. For instance, Fluctuation Strength (High bin) yields a gain of 53.8× with a 95% CI of [46.8×, 61.9×], while Loudness (Mid bin) produces a gain of 14.8× with a 95% CI of [12.8×, 16.7×]. One-sided bootstrap hypothesis tests (H_0_: Gain ≤ 1) return *p* < 0.001 for all metrics.

These results confirm that perceptual dispersion remains statistically and practically significant even under strict equal-energy constraints. Although averaging across heterogeneous aircraft types reduces absolute CV values compared with the single-aircraft case, the relative divergence between perceptual metrics and energy remains robust.

Overall, the persistence of dispersion gain across all bins demonstrates that auditory-model-based descriptors retain dimensions of variation that are systematically compressed when aircraft noise events are represented solely by integrated energy quantities.

To evaluate robustness against outlier filtering, the multi-dimensional IQR rejection factor was varied from 0.5× to 2.0×, resulting in retained sample sizes ranging from 487 to 695 events. As shown in Table 7, the dispersion gain of psychoacoustic metrics remained consistently large across all configurations.

The minimum gain observed among all descriptors and energy bins ranged from 8.24× (IQR = 0.5×) to 8.81× (IQR = 1.5×). Importantly, the minimum lower bound of the 95% bootstrap confidence interval remained between 7.13× and 7.83× across all IQR settings. In every case, the confidence intervals did not cross unity, and all one-sided bootstrap tests rejected the null hypothesis Gain ≤ 1 (*p* < 0.001).

Although stricter IQR filtering reduces sample size, the magnitude and statistical significance of dispersion gain remain stable. These results confirm that the observed perceptual variability is not driven by specific outlier rejection choices but represents an intrinsic structural property of the dataset.

### 3.5. Temporal Consistency and Divergence Between SPL and Sound Quality Metrics

**Figure 7:**
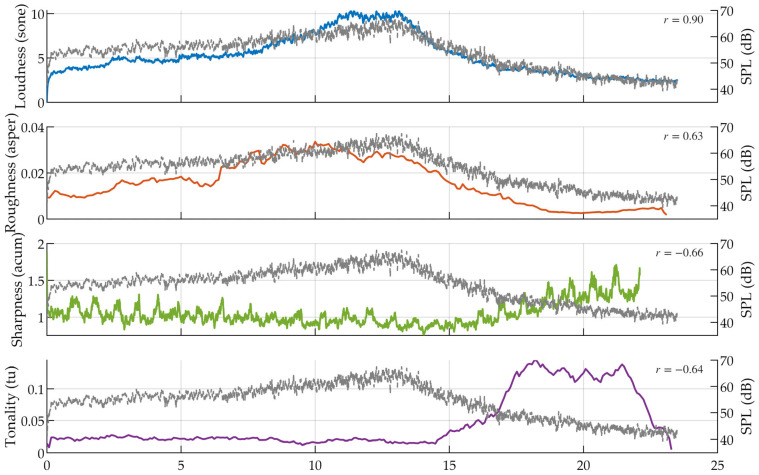
Time-varying sound quality metrics of aircraft noise compared with Sound Pressure Level (SPL) profiles. From top to bottom, the subplots illustrate the trends of Loudness, Roughness, Sharpness, and Tonality (colored solid lines) relative to the SPL (gray dashed line). The Pearson correlation coefficient in each subplot indicates the degree of consistency between the specific SQM and the overall energy evolution.

Figure 7 presents the temporal evolution of several psychoacoustic descriptors during a representative flyover event and compares them with the corresponding sound pressure level (SPL) profile.

At a global scale, loudness generally follows the overall trend of SPL, as reflected by the high correlation coefficient (r=0.90). This behavior is expected because loudness is strongly influenced by total acoustic magnitude. Nevertheless, minor deviations can still be observed around the rising and falling edges of the event, indicating that spectral distribution and auditory weighting also contribute to its dynamics.

In contrast, roughness shows a weaker correspondence with the SPL contour (r=0.63). Although the metric increases during the approach phase, its decline occurs earlier than the SPL peak, suggesting that modulation-related characteristics evolve differently from energy accumulation.

Sharper discrepancies appear in sharpness and tonality. For sharpness (r=−0.66), elevated values occur in later segments of the event where the SPL has already decreased. This indicates a shift in spectral emphasis toward higher-frequency components rather than simple dependence on overall level.

Tonality (r=−0.64) exhibits the most pronounced independence from the energy envelope. Distinct peaks emerge during the trailing phase, implying that tonal prominence may become more noticeable even as total intensity diminishes.

Taken together, these observations suggest that while some perceptual descriptors retain partial correlation with energy variation, they also reveal temporal structures that are not strictly governed by the SPL trajectory. This intra-event behavior aligns with the statistical findings presented earlier, where perceptual variability persists under constrained energy conditions.

## 4. Discussion

The results obtained in this study provide several complementary perspectives on how aircraft noise characteristics are represented under continuous monitoring conditions. By integrating spatiotemporal event matching, stratified data validation, manifold visualization, and equal-energy constrained analysis, the findings collectively illustrate the structural differences between traditional energy-based descriptors and perceptually motivated acoustic features.

### 4.1. Interpretation of Energy-Perception Representation Differences

Energy-based metrics such as $L_{max}$, $L_{SE}$, and related exposure indicators remain effective descriptors for representing the overall magnitude of acoustic emissions. Their statistical association with aircraft configuration parameters—particularly thrust and bypass ratio—confirms that these indicators largely reflect macroscopic physical characteristics of propulsion systems. In practical monitoring systems, such scalar quantities therefore provide a stable basis for regulatory reporting and long-term environmental noise statistics.

However, the analyses performed in this study indicate that energy descriptors capture only a limited subset of the acoustic variability present in operational aircraft noise. When events are examined under constrained energy intervals, perceptual descriptors continue to exhibit substantial dispersion. The equal-energy analysis shows that the coefficient of variation of psychoacoustic metrics exceeds that of $L_{SE}$ by one order of magnitude or more, with dispersion gains ranging from approximately 8× to over 50× depending on the metric. These results demonstrate that signals with nearly identical integrated energy can still exhibit substantial differences in spectral balance, temporal modulation, and tonal structure.

Such divergence highlights a fundamental limitation of scalar energy representations. Energy integration compresses the multidimensional spectral–temporal structure of aircraft noise into a single amplitude-dominated parameter. As a result, variations associated with modulation behavior, harmonic prominence, and auditory masking processes are largely suppressed.

### 4.2. Mechanisms Behind the Observed Physical–Perceptual Decoupling

A notable observation in the correlation analysis is the weak relationship between aircraft configuration parameters and psychoacoustic descriptors. While parameters such as bypass ratio show consistent correlations with energy metrics, their associations with perceptual quantities—including roughness, sharpness, and tonality—remain sparse and comparatively weak.

This apparent decoupling does not imply that aircraft design has no influence on perceptual sound characteristics. Instead, it reflects the multi-stage transformation between source generation, propagation, and auditory perception.

First, propulsion configuration primarily determines the total acoustic power radiated by the aircraft. Parameters such as thrust level, fan diameter, and bypass ratio directly influence jet turbulence noise and fan tonal components, which explains their strong relationship with integrated energy indicators.

Second, the spectral and modulation structure perceived at the monitoring point is shaped by several additional processes that are not directly encoded in global aircraft design variables. These include:

- Propagation effects, such as Doppler shift, atmospheric absorption, and ground reflections, which dynamically reshape spectral energy distribution during the flyover.
- Operational variability, including thrust setting, climb angle, and flight trajectory relative to the monitoring station.
- Temporal modulation mechanisms, arising from rotor–stator interaction tones and aerodynamic turbulence fluctuations.

Because psychoacoustic metrics are derived from auditory models incorporating critical-band filtering, temporal integration, and masking effects, they are sensitive to these fine structural variations. Consequently, perceptual descriptors reflect a combination of source physics and propagation-induced modulation processes rather than simple scaling with aircraft design parameters.

This interpretation is consistent with the manifold visualization results. The UMAP embeddings demonstrate that energy descriptors organize the dataset along a dominant amplitude gradient, whereas psychoacoustic features reveal additional orthogonal axes of variation associated with spectral and modulation characteristics. These additional dimensions correspond to perceptual attributes that are not linearly governed by macroscopic aircraft configuration parameters.

### 4.3. Practical Deployment and Computational Considerations for Continuous Monitoring

Beyond the analytical differences between energy-based indicators and psychoacoustic descriptors, an important question concerns the practical feasibility of deploying such perceptual feature extraction within continuous aircraft noise monitoring systems. Because unattended monitoring terminals operate for extended periods and must process large volumes of acoustic data, computational efficiency and storage requirements become critical considerations for real-world implementation.

In the proposed framework, psychoacoustic feature extraction is based on standard auditory modeling procedures, including short-time spectral analysis, critical-band filtering, and temporal modulation analysis. These operations rely primarily on fast Fourier transform (FFT) processing and filterbank operations, which scale approximately linearly with signal length. For typical aircraft noise events with durations of approximately 40–50 s and a sampling rate of 48 kHz, the computational workload remains moderate and can be efficiently handled by embedded computing platforms commonly used in environmental monitoring systems. In practical tests, the processing time required to compute the complete set of psychoacoustic descriptors is in the order of seconds on standard single-board computers, indicating that the computational burden is compatible with operational monitoring pipelines.

Furthermore, continuous psychoacoustic analysis does not necessarily require strict real-time processing of the entire audio stream. In ISO 20906-compliant monitoring systems, acoustic data processing is typically event-driven, meaning that detailed feature extraction is triggered only after a valid aircraft noise event has been detected. Because the temporal spacing between consecutive aircraft movements in typical runway operations often exceeds several tens of seconds, sufficient processing time exists between events to perform psychoacoustic calculations without interfering with the acquisition of subsequent measurements. This event-based processing strategy significantly reduces the effective computational load compared with continuous full-stream analysis.

Data storage requirements also remain manageable within this framework. Rather than storing high-resolution waveform data for long-term analysis, the monitoring pipeline retains compact feature descriptors derived from the acoustic signals. The dimensionality of the psychoacoustic feature vectors is several orders of magnitude smaller than that of the original audio recordings, enabling efficient storage and long-term archival of monitoring data. In addition, the reduced data volume facilitates large-scale statistical analysis and cross-event comparisons without imposing excessive storage overhead.

Taken together, these considerations indicate that the integration of psychoacoustic feature extraction into continuous aircraft noise monitoring systems is computationally feasible and operationally practical. The proposed framework can be incorporated into existing monitoring infrastructures with limited additional processing or storage requirements, while providing enhanced analytical resolution for characterizing aircraft noise beyond conventional energy-based indicators. Future work may further investigate which quantitative descriptors are most suitable for real-time monitoring applications, with particular emphasis on identifying a compact subset of perceptually informative indicators that balance computational efficiency, interpretability, and monitoring robustness.

## 5. Conclusions

This study presented a data-driven investigation of aircraft noise characteristics based on real-world continuous monitoring data collected at an operational airport environment. A complete analytical workflow was established, including spatiotemporal association between acoustic events and ADS-B trajectories, stratified data validation using model-specific IQR filtering, and multidimensional feature extraction based on psychoacoustic models. This framework enables the systematic analysis of aircraft noise characteristics beyond conventional energy-based monitoring approaches.

The comparative analyses reveal several key findings. First, traditional energy-based indicators effectively describe the overall magnitude of aircraft noise and show clear associations with macroscopic aircraft configuration parameters such as thrust and bypass ratio. However, these scalar descriptors tend to compress the spectral–temporal structure of aircraft noise into a nearly one-dimensional representation dominated by amplitude scaling. Second, psychoacoustic descriptors—including loudness, roughness, sharpness, fluctuation strength, and tonality—retain significant variability even under strict equal-energy constraints. The dispersion of these perceptually motivated features substantially exceeds the residual variation of the controlling energy metric, indicating that aircraft noise events with similar exposure levels may still differ considerably in their fine spectral and modulation characteristics. Third, manifold visualization further demonstrates that different metric systems organize the same dataset in distinct ways: energy descriptors produce highly concentrated embeddings, whereas psychoacoustic features reveal a higher-dimensional structure that preserves additional perceptual information.

Overall, the results indicate that psychoacoustic representations provide complementary dimensions of information beyond integrated energy levels. Integrating perceptually grounded descriptors into continuous aircraft noise monitoring systems therefore has the potential to enhance the analytical resolution and interpretability of monitoring data, particularly in situations where conventional metrics show limited discriminative capability.

Future research may further expand this framework in several directions. First, multi-site monitoring datasets could be incorporated to evaluate the generalizability of the proposed methodology under different airport configurations and operational conditions. Second, integrating psychoacoustic features with subjective response data and annoyance studies would help clarify the perceptual relevance of the extracted descriptors. Finally, further work may focus on identifying a compact subset of perceptually informative indicators that are most suitable for real-time monitoring applications, balancing computational efficiency, interpretability, and robustness in large-scale operational monitoring systems.

## Figures and Tables

**Table 1:** Precision–Recall Performance under Different Spatiotemporal Matching Configurations.

±Time (s)	Angle (°)	Retained	True Positive	False Positive	False Negative	Precision (%)	Recall (%)
30	15	874	840	34	216	96.1	79.5
30	30	959	920	39	136	95.9	87.1
30	45	1009	960	49	96	95.1	90.9
45	15	944	915	29	141	96.9	86.7
45	30	1037	1005	32	51	96.9	95.2
45	45	1091	1030	61	26	94.4	97.5
60	15	980	960	20	96	98.0	90.9
60	30	1078	1056	22	0	97.9	100
60	45	1135	1056	79	0	93.0	100
90	15	1066	1000	6	56	99.4	94.7
90	30	1110	1056	54	0	95.1	100
90	45	1170	1056	114	0	90.3	100

**Table 2:** Basic Model Information.

AirType	MTOM (kg)	MLM (kg)	Engine	Wingspan	Fuselage Length	BPR	Thrust
A320-214	78,000	66,000	CFM56-5B4/3	34.1	37.57	5.9	27,000
A320-232	78,000	66,000	V2527-A5	34.1	37.57	4.8	26,600
A321-211	93,500	77,800	CFM56-5B3/3	34.1	44.51	5.4	33,000
A321-231	93,500	77,800	V2533-A5	34.1	44.51	4.5	33,000
A320-271N	79,000	67,400	PW1127G1-JM	35.8	37.57	12.5	27,000
A320-251N	79,000	67,400	LEAP-1A26E1	35.8	37.57	11	27,000
B737-79L	70,080	58,604	CFM56-7B	35.8	33.63	5.3	22,700
B737-81B	79,000	67,400	CFM56-7B	35.8	39.47	5.1	26,300
B737-89L	79,000	67,400	CFM56-8B	35.8	39.47	5.1	26,300
B737-85C	79,000	67,400	CFM56-9B	35.8	39.47	5.1	26,300
B737-89P	79,000	67,400	CFM56-10B	35.8	39.47	5.1	26,300
B737-8MAX	82,191	69,308	LEAP-1B	35.92	39.47	9	28,000
190LR	51,800	44,000	CF34-10E5	28.72	36.24	5.4	18,500
195LR	52,290	45,800	CF34-10E7	28.72	38.65	5.4	20,000

**Table 3:** Intrinsic dimensionality and variance concentration of the two feature spaces.

Feature Space	PCA Top-2 Explained Variance	Participation Ratio Dimension
Energy metrics	98.38%	1.20
Psychoacoustic metrics	54.98%	4.62

**Table 4:** UMAP neighborhood stability across hyperparameter configurations.

Feature Space	Mean Jaccard Similarity	Std
Energy metrics	0.766	0.027
Psychoacoustic metrics	0.706	0.033

**Table 5:** Statistical validation of psychoacoustic dispersion under equal-energy constraint (B737-89L).

Metric	N (Low/Mid/High)	CV_LSE (%)	CV_Metric (%) [95%CI]	Gain [95%CI]	*p*-Value
Loudness	24/56/23	0.60–0.66	10.8–18.5 [5.4–24.8]	18.1–28.1 [8.5–35.6]	<0.001
Roughness	24/56/23	0.60–0.66	10.2–13.0 [5.5–15.8]	15.4–20.9 [8.3–26.9]	<0.001
Sharpness	24/56/23	0.60–0.66	6.6–11.6 [4.7–14.4]	11.1–17.5 [7.6–30.2]	<0.001
Fluctuation Strength	24/56/23	0.60–0.66	42.3–44.7 [31.1–56.2]	64.1–74.6 [46.5–132.3]	<0.001
Tonality	24/56/23	0.60–0.66	20.8–23.3 [11.5–27.9]	31.6–37.6 [18.8–67.5]	<0.001

**Table 6:** Bootstrap-based dispersion comparison under equal-energy constraints (full dataset).

Metric	Bin	N	CV_LSE(%)	CV_Metric(%)	Gain [95%CI]	*p*-Value
Loudness	Low	43	1.24	32.68	26.32 [21.44–32.64]	<0.001
Loudness	Mid	407	1.21	17.89	14.75 [12.78–16.72]	<0.001
Loudness	High	195	0.87	17.82	20.60 [16.30–25.62]	<0.001
Roughness	Low	73	1.24	22.89	18.43 [14.97–22.89]	<0.001
Sharpness	Mid	407	1.21	10.39	8.57 [7.81–9.42]	<0.001
Fluctuation Strength	High	195	0.87	46.53	53.79 [46.75–61.91]	<0.001
Tonality	High	195	0.87	26.30	30.40 [26.27–36.41]	<0.001

**Table 7:** Robustness of dispersion gain under varying IQR rejection factors.

IQR Factor	N	Min Gain	Min 95% CI Lower Bound	%CI > 1
0.5	487	8.24	7.13	100%
1.0	627	8.52	7.45	100%
1.5	678	8.81	7.83	100%
2.0	695	8.76	7.70	100%

## Data Availability

The data can be provided on request.

## Supplementary Materials

The following supporting information can be downloaded at: https://www.mdpi.com/article/10.3390/s26061842/s1, Table S1 Stability of UMAP embeddings under different parameter configurations evaluated by Jaccard similarity.
