# Supplementary material for: A Psychoacoustic Feature Extraction and Spatio-Temporal Analysis Framework for Continuous Aircraft Noise Monitoring
Source: Sensors (Basel). 2026 Mar 14;26(6):1842. doi: 10.3390/s26061842 (PMC13030723; doi:10.3390/s26061842)
Supplement: Supplementary file 1 [file sensors-26-01842-s001.zip › sensors-4175360-supplementary.pdf]

Supplementary

Table S1. Stability of UMAP embeddings under different parameter configurations evaluated by Jaccard similarity.

N_neighbors	Min_dist	Jacc_trad	Jacc_sqm
10	0.05	0.7481	0.6757
15	0.05	0.8032	0.7458
20	0.05	0.7934	0.7407
25	0.05	0.7646	0.6921
30	0.05	0.7642	0.6919
10	0.1	0.7476	0.7224
15	0.1	0.8144	0.7486
20	0.1	0.7800	0.7325
25	0.1	0.7586	0.6803
30	0.1	0.7546	0.6895
10	0.2	0.7779	0.7201
15	0.2	0.7914	0.7413
20	0.2	0.7306	0.7017
25	0.2	0.7373	0.6705
30	0.2	0.7209	0.6372
